# Mining for diagnostic information in body surface potential maps: A comparison of feature selection techniques

**DOI:** 10.1186/1475-925X-4-51

**Published:** 2005-09-02

**Authors:** Dewar D Finlay, Chris D Nugent, Paul J McCullagh, Norman D Black

**Affiliations:** 1 School of Computing and Mathematics, Faculty of Engineering, University of Ulster, Shore Road, Belfast, UK

## Abstract

**Background:**

In body surface potential mapping, increased spatial sampling is used to allow more accurate detection of a cardiac abnormality. Although diagnostically superior to more conventional electrocardiographic techniques, the perceived complexity of the Body Surface Potential Map (BSPM) acquisition process has prohibited its acceptance in clinical practice. For this reason there is an interest in striking a compromise between the minimum number of electrocardiographic recording sites required to sample the maximum electrocardiographic information.

**Methods:**

In the current study, several techniques widely used in the domains of data mining and knowledge discovery have been employed to mine for diagnostic information in 192 lead BSPMs. In particular, the Single Variable Classifier (SVC) based filter and Sequential Forward Selection (SFS) based wrapper approaches to feature selection have been implemented and evaluated. Using a set of recordings from 116 subjects, the diagnostic ability of subsets of 3, 6, 9, 12, 24 and 32 electrocardiographic recording sites have been evaluated based on their ability to correctly asses the presence or absence of Myocardial Infarction (MI).

**Results:**

It was observed that the wrapper approach, using sequential forward selection and a 5 nearest neighbour classifier, was capable of choosing a set of 24 recording sites that could correctly classify 82.8% of BSPMs. Although the filter method performed slightly less favourably, the performance was comparable with a classification accuracy of 79.3%. In addition, experiments were conducted to show how (a) features chosen using the wrapper approach were specific to the classifier used in the selection model, and (b) lead subsets chosen were not necessarily unique.

**Conclusion:**

It was concluded that both the filter and wrapper approaches adopted were suitable for guiding the choice of recording sites useful for determining the presence of MI. It should be noted however that in this study recording sites have been suggested on their ability to detect disease and such sites may not be optimal for estimating body surface potential distributions.

## Background

Although extensively utilised, the limitations of the conventional 12-lead ECG for optimal detection of cardiac abnormalities are widely appreciated [1]. The main deficiency in the 12-lead approach is the fact that only 6 chest electrodes are incorporated which cover a relatively constrained area of the precordium. The main reason for the choice of the location of the conventional precordial electrodes, suggested by Wilson over 70 years ago [2,3], was the need to adopt some standard which to this day has remained relatively unchallenged. In the years since then, the growing appreciation for the limitations of the conventional precordial electrode positions and the increase in understanding of the localisation of various cardiac abnormalities on the body surface has led to the suggestion of various alternatives. One of the most widely studied alternatives to the 12-Lead ECG in both clinical and experimental electrocardiology has been the BSPM. In this approach, anything between 32 and 219 electrodes [4] are used in an attempt to sample all electrocardiographic information as projected onto the body's surface. The merits of this enhanced spatial sampling are obvious, in that, localised abnormalities that are perhaps difficult to detect using the 12-lead approach can readily be picked up with the additional electrodes. As well as this ability to provide more diagnostic information, BSPMs facilitate an alternative method for visualisation as recorded data can be displayed as a sequence of contour maps, allowing isolation of significant electrocardiographic events in both space and time.

In body surface potential mapping, many of the inadequacies associated with the conventional 12-lead approach are addressed, but despite this, clinical utilisation outside the research laboratory is close to negligible. Several reasons exist for this lack of clinical uptake. Kors *et al*. [5] suggests the opposing interests of two groups as the main motivating factor: on one hand researchers are keen to further the diagnostic potential of the ECG using all information on the body surface, on the other hand, clinicians satisfied with the standard 12-lead ECG are reluctant to replace it with one of perceived technological complexity. This complexity stems from the requirement to sample dozens of channels of ECG information simultaneously, with the application of the associated number of chest electrodes viewed as highly impractical, particularly in acute care.

To address the impracticalities associated with high density spatial sampling procedures, investigators are interested in exploiting the redundancy in BSPMs to suggest 'limited lead' systems. This process can be succinctly described as locating the minimum number of recording sites required to capture the maximum amount of ECG information [6]. Although in all studies the number and location of sites is the focus, there are two main ways in which 'optimality' can been quantified. These are:

1. Sites that provide the maximum diagnostic information allowing enhanced discrimination between abnormalities.

2. Sites that allow the most accurate estimation of the heart's activity at other sites where information has not been recorded.

The first significant work on this problem of selecting limited leads was conducted by Barr *et al*. [7] who proposed a technique based on principal component analysis to locate 24 recording sites that allowed consistent representation of the total body surface potential during depolarisation (over the QRS). Subsequent to this, and based on a more representative dataset, Lux *et al*. [8,9] analysed correlation and covariance in 192 lead BSPMs to suggest 32 optimal recording sites that could be used to reconstruct the original BSPM frames with a level of error that was consistent with the estimated system noise, which, in the studied dataset was estimated to be 20 μV. In both sets of studies [7-9], the recording sites were chosen based on the ability to estimate potentials at sites that were not recorded. Kornreich *et al*. [10-12] on the other hand conducted several studies that suggested electrode configurations that were most suitable for diagnosing a range of abnormalities; in each study the objective was to find the best discriminating recording sites. More recently Kors *et al*. [5] conducted a study where the positions of the 6 precordial electrodes in the conventional ECG were altered intuitively to provide greater information capture. In this study, it was found that two of the standard precordial leads could be re-positioned to provide greater information capture whilst the remaining precordial electrodes could accurately reconstruct the 12-lead ECG. Although well validated in the research literature, the lead systems proposed by all of these studies have never been widely accepted in clinical practice.

Regardless of the rationale for the choice of recording sites, early investigators were limited to mathematical and statistical techniques in the selection process, exploiting phenomena such as correlation and variance in the recorded signals. Although yielding acceptable results, new techniques that have emerged through the proliferation of domains such as data mining and knowledge discovery [13] may provide greater insight into the process of lead selection. The process of lead selection is itself analogous to that of 'feature selection' which is a term commonly used in the aforementioned domains of data mining and knowledge discovery to describe the elimination of redundant variables in a dataset [14]. Ideally, feature selection would involve exhaustively evaluating all possible combinations of input features and choosing the best subset, but in reality the computational cost of this is prohibitive. For this reason, much research effort has been directed at developing algorithms and strategies that locate optimal features at low computational cost. In the current study commonly used feature selection methodology has been applied to the BSPM domain in order to select electrode subsets that are best for discriminating between normal subjects and those with MI.

In many domains, determining the features or variables that are relevant can give a useful insight into the nature of the prediction problem at hand [15]. This is particularly the case in the BSPM domain as information on the location of the best recording sites may complement information or understanding for those interested in the underlying cardiac behaviour. To provide both information that could be used to better understand cardiac function as well as information that is useful for computerised classification, two common feature selection techniques, 'filters' and 'wrappers', were employed in the current study.

### Filters

In the filter approach, the predictive performance of each individual feature is assessed and features that are deemed unnecessary are 'filtered' out [16]. The resulting measure of performance for each feature is used in a ranking process and the desired number of features *n*, with the highest scores, are selected. Although in this description, and throughout the current study, consideration is given to features on an individual basis only, subsets of features can also be considered for inclusion or elimination. The main evaluation techniques include correlation analysis (determining how each variable correlates with the target variable) and discrimination analysis (determining how each variable acts as a classifier). This process is often described as classifier independent as although a single variable classifier may be used in the selection of each feature, the selected feature subset as a whole is not chosen to suit any classifier.

### Wrappers

In this approach, the aim is to find the best subset of features by testing them with the classifier that they are intended for use with. As well as picking features to suit the classifier, this approach is advantageous in that features are selected based on how well they work together with other features. The process begins with the suggestion of a feature subset which is evaluated using the desired classifier, this feature subset is repeatedly modified (features are added and/or removed) and evaluated until the desired level of performance is attained. The two most common methods used to generate the feature subsets are SFS and Sequential Backward Elimination (SBE) [17]. In SFS the process begins with an empty set of features. In the first iteration, all feature subsets containing only one feature are evaluated i.e. the performance of each individual feature is evaluated. The feature with the highest accuracy is used as the basis for the next iteration, where, each remaining feature is evaluated in conjunction with the previously selected feature. The feature that performs best in conjunction with the first feature is selected forming the basis for the next iteration. This process is repeated until all features have been incorporated in the subset, or the desired level of accuracy has been reached. In SBE the process begins with a subset that contains all available features, from which features are removed one by one based on the performance of the remaining features. In theory, going backwards from the full set of features in the data may capture interacting features more easily; however this is at greater computational expense as building classifiers where there are few features in the dataset is much faster [18]. At this point it is worth underlining that the main difference between the filter and wrapper approach is that, in the filter approach, no feedback is used from the classification model.

## Methods

**Figure 1:**
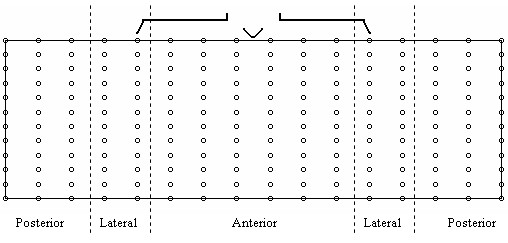
**192 Electrode Array**. Schematic representation of the 192 electrode array, depicted as an unrolled cylindrical matrix. The middle region correspond with the anterior torso and the left and right regions correspond with the posterior.

**Figure 2:**
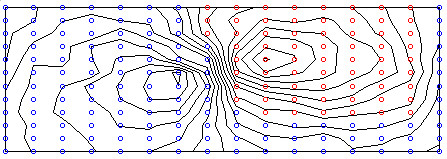
**Example QRS isointegral map**. Example of a QRS isointegral map derived from subset with Inferior wall MI (IMI). The areas of positive and positive and negative polarity are indicated with red and blue circles respectively.

From a practical perspective, the most widely used technique in locating recording sites or lead subsets is to start off with a dataset recorded using a full BSPM electrode array and try to find the best recording sites in that array that satisfy the desired criteria. The locations of these 'best sites', which are a subset of the original BSPM array, are then proposed as the new limited lead set. In the current study, BSPMs recorded using an electrode array of 192 electrodes were used. This dataset is of similar genre in terms of acquisition format as that used in [8,9]. The layout of the electrodes is depicted in the schematic in Figure 1. In this configuration electrodes are placed in equally-spaced columns, each consisting of 12 electrodes, around the thoracic circumference. For each subject, the 192 channels of ECG information were sampled simultaneously at 1000 Hz for a duration of several seconds. Subsequent to recording, this data was reduced to represent just one cardiac cycle which on average consisted of 600 milliseconds of information. This was achieved through RMS averaging. In all, maps recorded from 116 subjects were studied. Within this group of subjects, 59 were considered as normal exhibiting no disease symptoms, the remaining 57 had electrocardiographic evidence of old MI with lesions at various locations. Table 1 describes the composition of the dataset with a breakdown of the various infarct locations. Following recording, the map sequences from each patient were processed to provide QRS, STT, and QRST isointegral features. This is common practice in the BSPM domain and involves reducing a sequence of map frames to one single map. Often referred to as isoarea mapping, the process involves summing all the potentials under a specific portion of the ECG trace at each recording site, and plotting a contour map using the resulting values. In this study, QRS and STT isointegrals were calculated to illustrate mean potential distributions during depolarisation (activation) and repolarisation (recovery) respectively, and QRST isointegrals were calculated to illustrate mean potential distributions during depolarisation and repolarisation combined. Each isointegral consists of 192 values which are used to generate a contour map. Figure 2 depicts an example of such a map, in this case a QRS isointegral map for a subject with Inferior wall MI (IMI). The pattern of extrema, maxima and minima of such a map are studied by the clinician in order to provide diagnosis and because the pattern is characterised by the 192 calculated values, these values can be used as inputs (features) to a computerised classifier. As we have calculated 3 such maps, we effectively end up with 576 features for each subject (3 × 192). This also translates to having 3 features per recording site per patient, e.g. for each recording site we have one QRS, one STT, and one QRST value. In all experiments, we combine the three set of isointegral measurements for each patient, resulting in 576 features per patient.

**Table 1:** Composition of dataset describing infarct locations.

Normals		**59**
Myocardial Infarction		**57**
Inferior	30	
Anterior	14	
Posterior	2	
Aterolateral	8	
Inferolateral	2	
Inferior-posterior	1	
Total		**116**

### Single variable classifier approach

In this study, the prediction accuracy of each individual feature was assessed using a Nearest Neighbour (NN) classifier [19]. From this point onwards we shall refer to this as the SVC approach as this is effectively a special case of the filter approach. Using Leave One Out (LOO) cross validation, the SVC was used to evaluate the performance of each individual feature on a randomly selected subset of 87 of the 116 original recordings. In LOO cross validation, the performance of the given feature is obtained by using all records bar one (86 recordings) to train the classifier and using the remaining record to evaluate the classifier. This process is repeated until every subset of one record is evaluated, the final performance being obtained through taking an aggregate of each individual classification outcome. The remaining 29 records were treated as a hold out set and were used to test the classification accuracy of various sets of the highest ranked features again in conjunction with a NN classifier. The same features were also tested using classifiers based on 5 Nearest Neighbour (5NN) and Logistic Regression (LR), this was performed to assess the chosen features on a range of classifiers.

### Sequential forward selection wrapper approach

**Figure 3:**
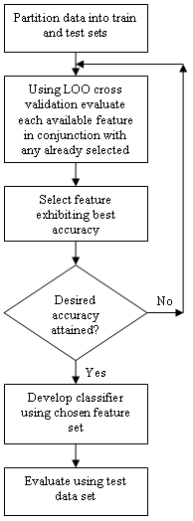
**Wrapper Based Feature Selection**. Illustration of the wrapper based feature selection approach where the feature subset is generated by iterative evaluation of available features.

In the current study, the high ratio of features to cases (observations) also becomes an issue in SBE, as most classifiers will exhibit instability when the number of features greatly exceeds the number of variables. For this reason the SFS approach was adopted, and applied using three separate classifiers (LR, NN, 5NN) which resulted in three feature sets each of which were classifier specific. In order to reduce the computational time associated with the procedure the SFS process was invoked for 100 iterations, resulting in selection of the 100 best features. To validate the results, the data set of 116 records was again randomly partitioned into a training set of 87 records and a test set of 29 records (same as above). The training set was then used to conduct the SFS process, and LOO cross validation was used on each iteration of the feature subset selection process. When the selection process was completed, the training set was used to develop a classifier using the selected features, which was then validated using the unseen test set. This approach was taken to alleviate optimistic biasing of the results as opposed to some studies, as pointed out by [18] that use the classification accuracy of the selection runs as a measure of final performance. A flow diagram illustrating the wrapper approach implemented in these experiments is shown in Figure 3.

**Figure 4:**
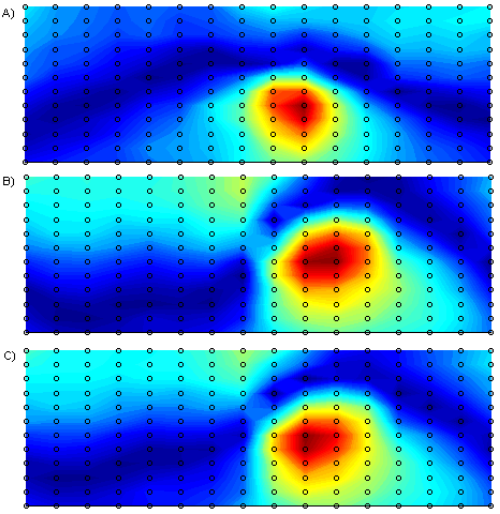
**Difference Maps**. Spatial representation of the absolute difference between the means of the normal and MI population for (a) QRS, (b) STT, and (c) QRST isointegrals. Magnitude of difference increases as the colour changes from dark blue through to dark red.

In addition to performing the experiments outlined above, a simple procedure was conducted to illustrate the localisation of difference between normal and MI populations in the training set. This procedure involved subtracting the mean of the normal population from the mean of the MI population and presenting the resulting difference as a contour map of the absolute values at each recording site. For each isointegral (QRS, STT, QRST) 'difference' maps were calculated and the results are illustrated in Figure 4. These difference maps have been generated merely for comparison with later results.

## Results

### SVC approach

**Figure 5:**
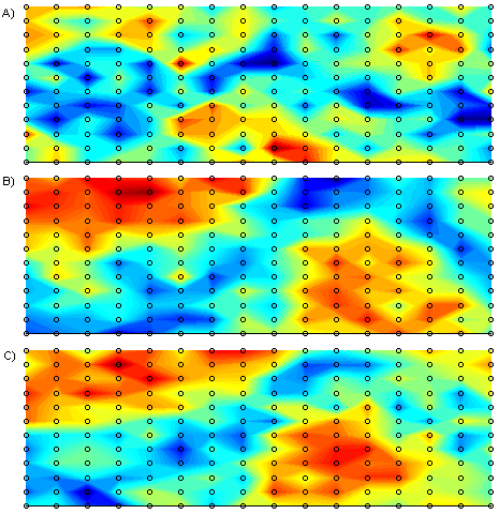
**Lead Performance Maps**. Spatial distributions of the prediction accuracy at each of the 192 recording sites for (a) QRS, (b) STT, and (c) QRST isointegrals. Magnitude of difference increases as the colour changes from dark blue through to dark red.

The classification accuracy obtained for each individual feature was used to rank the features in descending order of significance. In cases where two or more features had an identical score these features were ordered arbitrarily. A method of Lead Performance Maps (LPMs) similar to that adopted in [20] was used to graphically illustrate the spatial distribution of feature performance. In this approach a contour map is produced using the feature performance values positioned with the same layout of the original electrode array, the contour map is then shaded through linear interpolation between the 192 data points. In this study this resulted in three separate LPMs (for QRS, STT, QRST isointegrals) which are illustrated in Figure 5.

**Figure 6:**
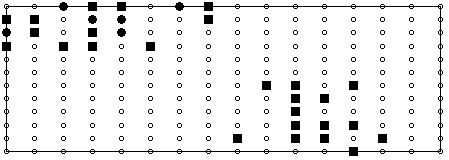
**Electrode Locations (SVC Approach)**. Locations of the 32 top recording sites as chosen using the variable ranking method. The first 6 sites chosen are highlighted using the filled circles; the filled squares show the remaining 26 sites.

As the overarching goal of this study was to assess the performance of limited electrode arrays the classification performance of the best 3, 6, 9, 12, 24, and 32 recording sites, deduced from the top ranked features, were evaluated. The choice of an upper limit of 32 is synonymous with the number of leads in the 'clinically practical' lead system proposed by Lux in [9]. It is worth pointing out that for each of the lead subsets it was possible to have more than the corresponding number of features as in some cases multiple features were selected for each lead. A schematic illustrating the positions of the top 32 recording sites is illustrated in Figure 6. For the sake of comparison with more conventional lead systems, the best 6 electrodes out of the 32 have also been highlighted. The classification accuracy of the various lead subsets is listed in Table 2. As well as classifications accuracies, measures for sensitivity and specificity have been included with all results.

**Table 2:** Subset Performance-SVC Approach. The performance of subsets of leads chosen using the SVC approach, evaluated using three classifiers.

No. Recording Sites	NN			5NN			LR		
	
	**ACC (%)**	SEN (%)	SPE (%)	**ACC (%)**	SEN (%)	SPE (%)	**ACC (%)**	SEN (%)	SPE (%)
3	**72.4**	85.7	80.0	**69.0**	64.3	73.3	**72.4**	78.6	66.7
6	**82.8**	71.4	93.3	**72.4**	64.3	80.0	**75.9**	78.6	73.3
9	**79.3**	85.7	73.3	**69.0**	71.4	66.7	**72.4**	78.6	66.7
12	**79.3**	78.6	80.0	**75.9**	78.6	73.3	**65.5**	78.6	53.3
24	**79.3**	78.6	80.0	**79.3**	71.4	86.7	**79.3**	85.7	73.3
32	**79.3**	78.6	80.0	**79.3**	71.4	86.7	**79.3**	85.7	73.3

### SFS approach

**Figure 7:**
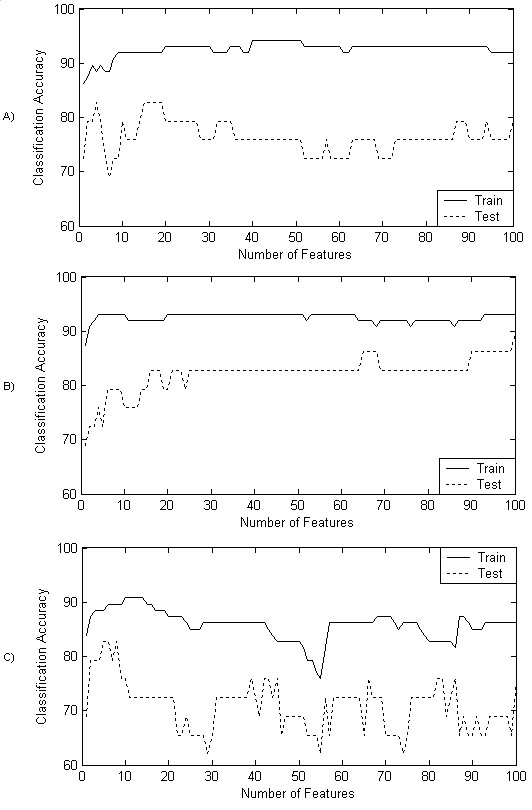
**Wrapper Performance**. Performance of the three SFS based wrappers using (a) NN classifier, (b) 5NN classifier, and (c) LR classifier. Each plot shows the classification accuracy as features are added during the selection process (train): And, the performance of selected features on an unseen test dataset (test).

**Figure 8:**
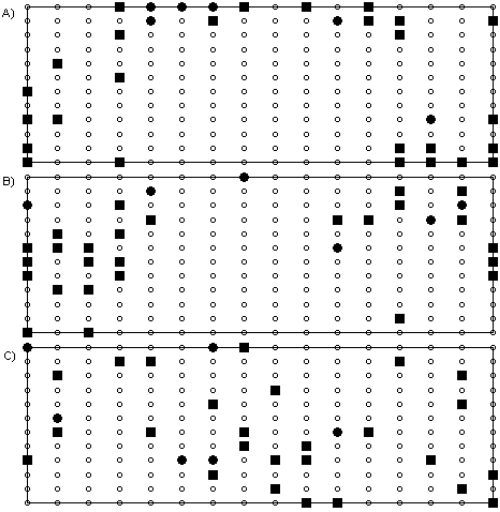
**Electrode Locations (SFS Approach)**. Recording sites chosen by SFS algorithms using (a) NN, (b) 5NN, and (c) LR. On each layout the top 32 recording sites have been illustrated. Additionally the top 6 sites which are a subset of the 32 are also shown (filled circles).

Because of the nature of the SFS approach, where the classification accuracy of each feature is determined accumulatively, it is not possible to display the individual classification accuracy of each feature using LPMs. What is possible, and probably of more significance from a raw computer science perspective, is the display of the performance of the SFS algorithm as features are accumulatively selected. This is depicted in Figure 7 where a graph for each wrapper based on a different classifier is shown. In these three graphs, the performance of the classifier in the feature evaluation and selection process is illustrated with the solid line, and the performance of the same classifier and feature set with unseen data is illustrated with the dashed line. In Figure 8, the best 32 leads chosen by each wrapper have been schematically represented as previously described, and as conducted for the filter approach the classification accuracy of the various lead subsets was also evaluated as presented in Table 3.

**Table 3:** Subset Performance-SFS Approach. Respective classification accuracy of recording sites chosen using NN, 5NN and LR wrappers.

No. Recording Sites	NN			5NN			LR		
	
	**ACC (%)**	SEN (%)	SPE (%)	**ACC (%)**	SEN (%)	SPE (%)	**ACC (%)**	SEN (%)	SPE (%)
3	**79.3**	71.4	86.7	**72.4**	71.4	73.3	**79.3**	85.7	73.3
6	**72.4**	71.4	73.3	**79.3**	78.6	80.0	**82.8**	92.9	73.3
9	**72.4**	50.0	93.3	**79.3**	78.6	80.0	**75.9**	92.9	60.0
12	**75.9**	64.3	86.7	**75.9**	78.6	73.3	**72.4**	85.7	60.0
24	**79.3**	78.6	80.0	**82.8**	85.7	80.0	**69.0**	78.6	60.0
32	**79.3**	71.4	86.7	**82.8**	85.7	80.0	**72.4**	71.4	73.3

## Discussion

The main observation from the preliminary difference map calculation illustrated in Figure 4 is that for all features per three maps there is significant localisation in the precordial region, indicating that on average these recording sites exhibit the most difference between normal and subjects with MI. Although this is useful in illustrating the significant localisation of difference between populations, this technique has limitations in lead selection as pointed out by Kornreich *et al*. [10], who suggested that the discriminant ability of each recording site is not truly reflected as intra-group variability is not taken into consideration.

The variable ranking procedure was primarily implemented to allow the selection of recording sites for evaluation in simple classification problems, and in this study the classification accuracy of 3, 6, 9, 12, 24, and 32 recording sites has been evaluated using three classifiers. From the results presented in Table 2, it can be observed that when using 24 recording sites, each classifier is capable of a predication accuracy of almost 80% which remained the same when the number of recording sites was increased to 32. Indeed, for all the configurations of recording sites evaluated, with the exception of one (12 sites with the LR classifier), a reasonable level of classification accuracy was observed. One characteristic of the results that deserves further explanation is the fact that the classification accuracy does not always increase, and sometimes decreases when the number of recording sites is increased. This is possibly due to the fact that in the selection procedure no consideration is given to the selection of variables that are correlated. In some classifiers correlation between variables can lead to deterioration in performance. It should also be noted that in some cases the seemingly coarse variations in performance, for example a change in 82.8% to 79.3% between 6 and 9 recording sites for the NN based SVC classifier, are largely attributable to the relatively modest size of the validation dataset. Evident in this particular example with a deterioration in performance of 3.5% through the misclassification of just one subject.

As well as providing useful information for classifier development this variable ranking also lends itself well to graphical display of the spatial distribution of the diagnostic features as is demonstrated in the LPMs in Figure 5. These LPMs show the areas of the torso where the classification accuracy of each individual feature is greatest. It can also be observed that the LPMs bear some resemblance to the difference maps that are displayed in Figure 4, in particular the corresponding maps for STT and QRST isointegrals bear significant resemblance with respect to the locations of areas of high and low significance.

In terms of the actual recording sites that were chosen using the SVC approach it can be seen that the upper right chest and back, and the lower left abdominal regions exhibit the highest concentrations of suggested sites. This of course is indicative of the information presented in the difference maps and the LPMs, however one interesting point is the fact that the six best electrodes are located in the upper right chest and back as opposed to the left precordial region as is the case with conventional electrocardiographic leads.

The performance of the three SFS algorithms depicted in Figure 7 gives a good indication of the general performance of this type of algorithm, as in general there is an increase in classification accuracy as features are initially added which is followed by a fluctuation, or decrease in accuracy as subsequent features are added. This is similar to the responses observed in other studies where non-electrocardiographic data has been used [15,21]. It can be observed that for all three wrappers, there is a consistent increase in performance on the train data as features are added, and in the NN and 5NN based approaches there is no significant deterioration in performance on the training data within the scope of the conducted experiments (up to 100 features). In the case of the LR based wrapper there is a less consistent response as performance deteriorates at approximately 20 features. This can be attributed to the fact that LR based classifiers exhibit significant instability when the number of variables is excessive compared with the number of cases. Although there is no definitive measure for predicting when this instability might occur, it can be seen here that 20 variables with 86 cases, a ratio of 10/43 induced this phenomenon. With regard to the performance associated with the test data, it can be seen that in some cases, particularly the NN based wrapper, there is not always consistent increase in performance as features are added. Again this is similar to observations in other studies [15,21] and appears to be a trait of the SFS approach

The inclusion of the sensitivity and specificity figures, as defined in [22], also provides some insight into how each set of features perform. These figures, which give an indication of how well subjects from each group (MI and Normal) have been correctly classified, were not taken into consideration during the selection process. The first observation is that the SFS selection approach using the 5NN classifier seems to provide the best balance between the two measures, as for each lead subset there is typically just a few percent difference between the measures. This is in contrast to some of the other selection/classifier combinations, for example the SFS/NN combination at 9 recording sites, where the sensitivity was 50% but the specificity was 93.3%. The reason for such an imbalance is likely to be attributable to the fact that these measures were not taken into consideration as features were being selected. If it were necessary to give preference to either sensitivity of specificity then consideration would have to be given to these variables during the selection process.

The classification accuracies obtained using the SFS wrappers (Table 3) show that this technique is as good as and in some cases better than the variable ranking procedure discussed above. In particular the performance of the 5NN classifier using features obtained from 24 and 32 recording sites is consistently better than the corresponding numbers of recording sites selected using the variable ranking approach. This can be attributed to two factors that make the wrapper approach superior in most applications. Firstly, as features are selected, their evaluation is based on how well they work together as a sub set, thus reducing the likelihood of selecting multiple features that are highly correlated and effectively measuring the same thing. Secondly, the characteristics of the final classifier are considered in the feature selection process therefore the chosen features are classifier specific. This second point accounts for the fact that although measuring sites were located on the same general areas of the torso as can be seen in Figure 8, there is significant discrepancy between the exact measuring sites chosen for each of the three wrappers. To illustrate just how specific the chosen recording sites are to each individual classifier a simple experiment was conducted. In this experiment the recording sites and hence features that were chosen by the 5NN based wrapper were tested on a LR classifier. The results are listed in Table 4. Here it can be seen that there is significant deterioration in the classification accuracy as the number of recording sites increases, illustrating that the features chosen for the 5NN classifier do not work anywhere as well for the LR classifier.

**Table 4:** Subset Performance-Cross Comparison. Classification accuracy of recording sites selected for 5NN classifier, evaluated on LR classifier.

No. Recording Sites	**ACC (%)**	SEN (%)	SPE (%)
3	**75.9**	85.7	66.7
6	**75.9**	85.7	66.7
9	**72.4**	85.7	60.0
12	**69.0**	85.7	53.3
24	**69.0**	85.7	53.3
32	**65.5**	78.6	53.3

**Figure 9:**
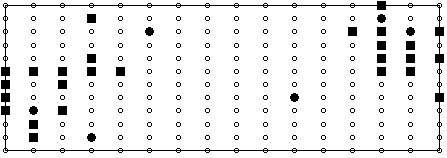
**Electrode Locations (Forced SFS Approach)**. Recording sites chosen by SFS algorithms using 5NN when the first selected feature is forced.

A further issue that we wished to investigate was the notion of 'uniqueness' introduced in [8]. In the study the authors suggested that there was no unique set of 'optimal leads' for reconstruction of BSPMs. Fuelled by the diversity of recording sites suggested in each of the techniques investigated in this study we wanted to find out if the same were true for recording sites that were selected for classification. To test for this condition, the experiment using the SFS wrapper based on the 5NN classifier was repeated. Under normal conditions this algorithm starts off by choosing the best individual feature as it makes its first pass through the list of available features. This time, however, the algorithm was forced to pick a non-optimal feature the first time around; subsequently the algorithm reverted back to normal operation choosing the best combinations from that point on. The 32 measuring sites chosen in this approach are illustrated in Figure 9, and the number of correctly classified cases are shown in Table 5. It can be seen that the pattern of chosen recording sites is not dissimilar to those for the original 5NN classifier based approach (Figure 8b), however, the exact location of each electrode differs in many cases, particularly within the case of the first 6 chosen electrodes as only one of these occupies the same position as in the original experiment. Consulting the table of classification accuracies it can be observed this rearrangement of electrodes does not necessarily come at a cost, indeed in the case of 9 and 12 recording sites a superior rate of classification is in fact achieved. These results would tend to suggest that like in the best recording sites chosen for reconstruction [8], there is no unique best set of electrodes for classification.

**Table 5:** Subset Performance-Forced SFS Approach. Classification accuracy of recording sites chosen using the 5NN based wrapper approach with a forced initial selection.

No. Recording Sites	**ACC (%)**	SEN (%)	SPE (%)
3	**79.3**	78.6	80.0
6	**75.9**	71.4	80.0
9	**86.2**	85.7	86.7
12	**86.2**	85.7	86.7
24	**82.8**	78.6	86.7
32	**75.9**	71.4	80.0

## Conclusion

In this study previously unutilised techniques have been applied to the perennial problem of selecting optimal recording sites from BSPMs. The filter and wrapper approaches to feature selection have been studied and have been found useful in locating useful diagnostic information. As well as evaluating the diagnostic capabilities of selected lead subsets, the study demonstrated how features chosen using the wrapper approach are specific to the given classifier. In addition, the study also introduced the notion of the non-uniqueness of selected lead subsets by suggesting two relatively different lead subsets using the same method, which exhibited comparable performance. Although the relatively small data set does limit the findings of the study, it is believed that the results obtained show that these techniques could be applied to a larger population of recordings to suggest limited lead systems that could be used in clinical practice.

A subtle limitation to the study is the fact that features used mainly in the BSPM domain are used to evaluate the classification performance of limited lead sets. These features are the isointegral measurements already mentioned. In reality, if a limited number of leads were used there may be a more effective way for representing the underlying ECG information, as isointegral maps were adopted mainly to counter the effect of excessive amounts of spatial data. This limitation does not prohibit the comparison of results within the study, however, it makes benchmarking with studies conducted by other investigators more difficult. To this end, if the study were to be extended it may be useful to investigate the use of temporal features such as wave amplitudes and durations as would be more common in conventional 12-lead electrocardiography.

Finally, the study has investigated the application of knowledge discovery to the problem of locating diagnostic information in BSPMs and the tools studied could be used to guide the development of recording system configurations. A clear distinction exists however between this approach and that where lead systems are developed to allow reconstruction of BSPMs. There is no evidence to suggest that recording sites selected based on their ability to discriminate between normal and abnormal are optimal for estimating potential distributions on the entire body surface, hence this area warrants further investigation.

## Authors' contributions

DDF was responsible for designing the study, conducting all experimental procedures, and along with CDN, interpreting and presenting the results. Both DDF and CDN participated equally in drafting the manuscript. PJM and NDB participated in the design of the study and also helped to draft the manuscript.
